# Supplementary figures and images for: Real-time reverse transcription loop-mediated isothermal amplification for rapid detection of SARS-CoV-2
Source: PeerJ. 2020 Jun 3;8:e9278. doi: 10.7717/peerj.9278 (PMC7275676; doi:10.7717/peerj.9278)

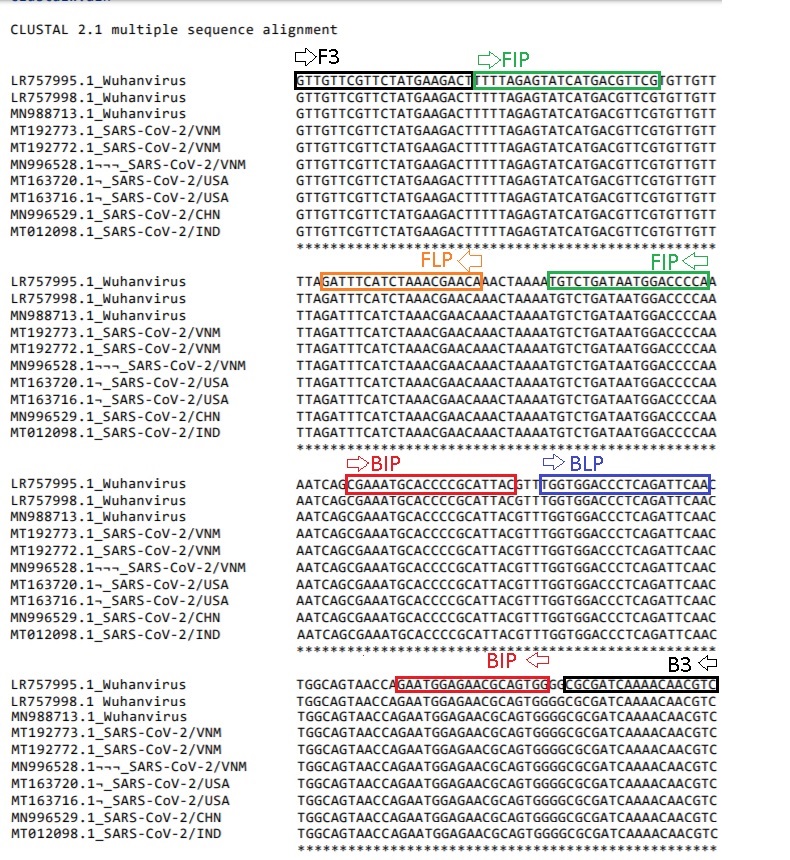

Supplement: Supplemental Information 1 — The sequences of the selected primers were conserved among the SARS-CoV2 sequences. [file peerj-08-9278-s001.jpg]

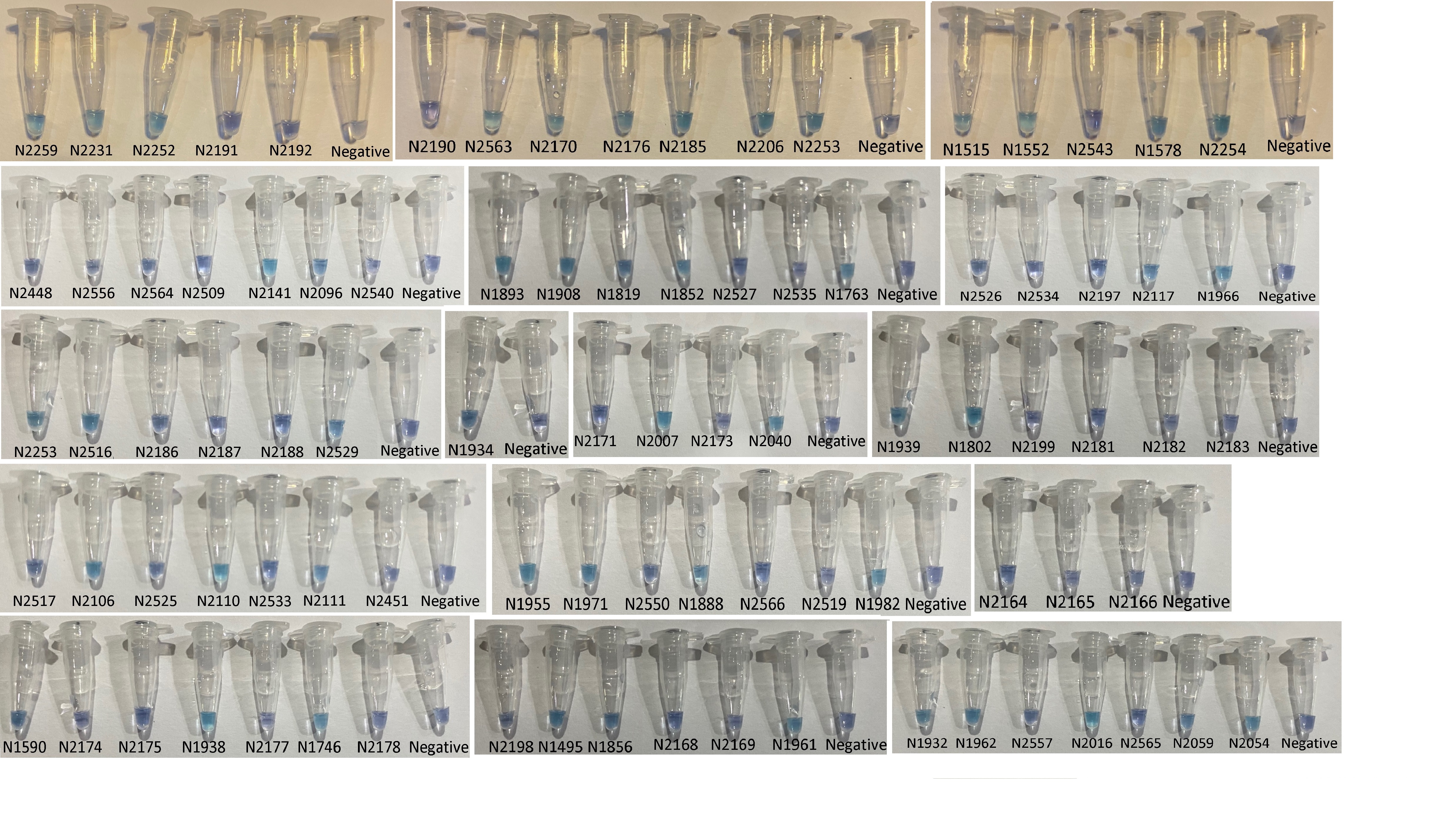

Supplement: Supplemental Information 3 — The RT-LAMP assay demonstrated a 100% sensitivity as all the 47 RNA samples that were positive by qRT-PCR were tested positive with RT-LAMP. None of the 42 qRT-PCR negative samples were positive for SARS-CoV2 using this assay. A positive reaction indicated by sky blue colour, while a violet colour indicates a negative reaction. [file peerj-08-9278-s003.jpg]

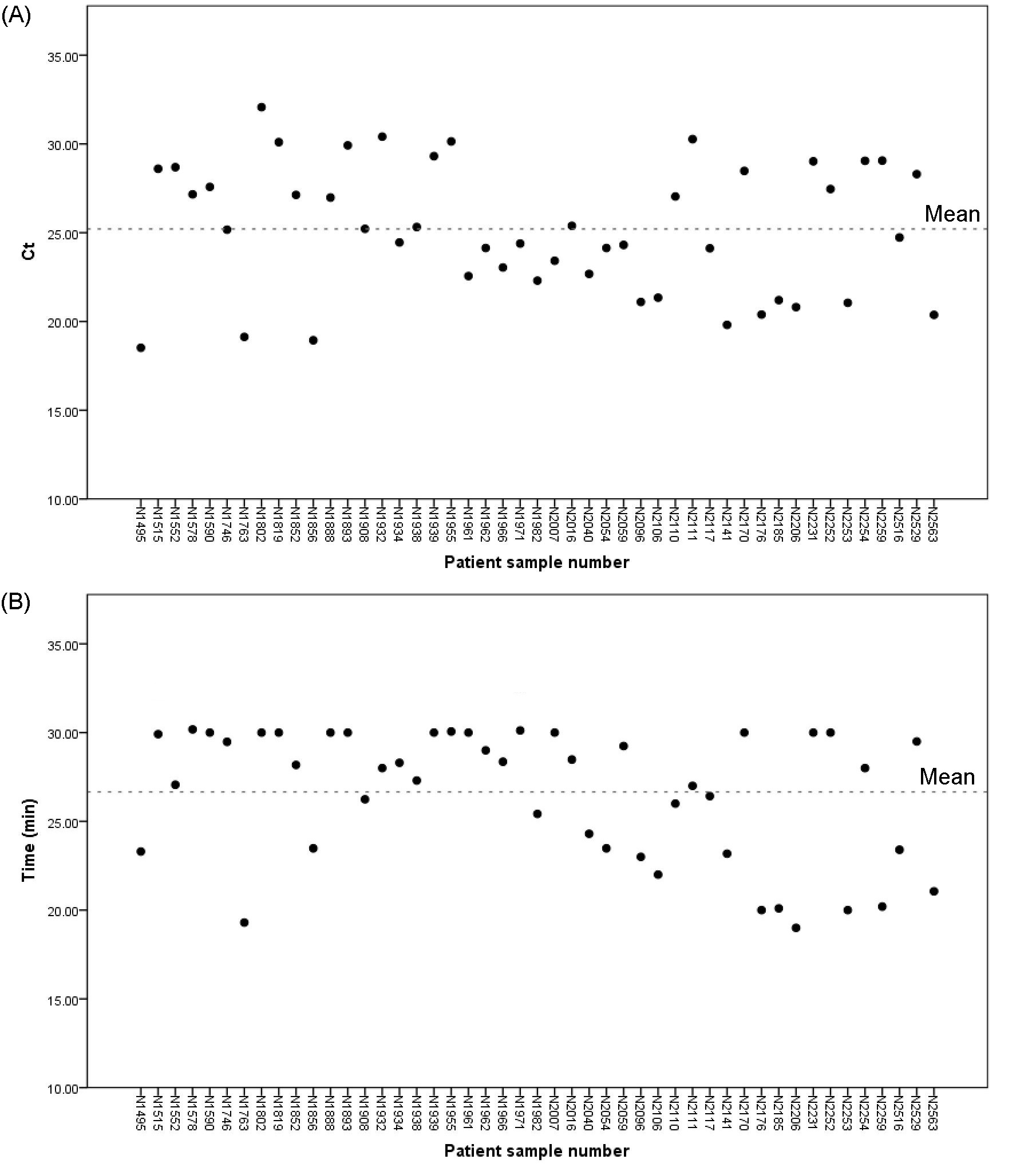

Supplement: Supplemental Information 4 — qRT-PCR threshold cycle values (A) and RT-LAMP reaction time (B) for all the samples used in this study. [file peerj-08-9278-s004.jpg]
