# Supplementary material for: Real-time reverse transcription loop-mediated isothermal amplification for rapid detection of SARS-CoV-2
Source: PeerJ. 2020 Jun 3;8:e9278. doi: 10.7717/peerj.9278 (PMC7275676; doi:10.7717/peerj.9278)
Supplement: Supplemental Information 2 [file peerj-08-9278-s002.pdf]

CLUSTALW Result

```
WARNING: possibly wrong combination

  --------------------------------
  Selected type :     PROTEIN
  Query sequence:     DNA
  --------------------------------
```

[clustalw.aln][clustalw.dnd][readme]

Select tree menu ▼ Exec

```
 CLUSTAL 2.1 Multiple Sequence Alignments


Sequence type explicitly set to Protein
Sequence format is Pearson
Sequence 1: LR757995.1_Wuhanvirus                  200 aa
Sequence 2: LR757998.1_Wuhanvirus                  200 aa
Sequence 3: MN988713.1_Wuhanvirus                  200 aa
Sequence 4: MT192773.1_SARS-CoV-2/VNM              200 aa
Sequence 5: MT192772.1_SARS-CoV-2/VNM              200 aa
Sequence 6: MN996528.1¬¬¬_SARS-CoV-2/VNM           200 aa
Sequence 7: MT163720.1¬_SARS-CoV-2/USA             200 aa
Sequence 8: MT163716.1¬_SARS-CoV-2/USA             200 aa
Sequence 9: MT012098.1_SARS-CoV-2/IND              200 aa
Sequence 10: MN996529.1_SARS-CoV-2/CHN              200 aa
Sequence 11: NC_004718.3_SARS_coronavirus           205 aa
Sequence 12: AY274119.3_SARS-related_coronavirus    205 aa
Sequence 13: MG772933.1_Bat_SARS-like_coronavirus   200 aa
Sequence 14: NC_014470.1_Bat_coronavirus            209 aa
Sequence 15: KU740200.1_MERS/Egypt                  207 aa
Start of Pairwise alignments
Aligning...



(Partial alignment)
Sequences (1:2) Aligned. Score: 41.5
(Partial alignment)
Sequences (1:3) Aligned. Score: 41.5
(Partial alignment)
Sequences (1:4) Aligned. Score: 41.5
(Partial alignment)
Sequences (1:5) Aligned. Score: 41.5
(Partial alignment)
Sequences (1:6) Aligned. Score: 41.5
(Partial alignment)
Sequences (1:7) Aligned. Score: 41.5
(Partial alignment)
Sequences (1:8) Aligned. Score: 41.5
(Partial alignment)
Sequences (1:9) Aligned. Score: 41.5
(Partial alignment)
Sequences (1:10) Aligned. Score: 41.5
(Partial alignment)
Sequences (1:11) Aligned. Score: 61.5
(Partial alignment)
Sequences (1:12) Aligned. Score: 61.5
(Partial alignment)
Sequences (1:13) Aligned. Score: 39.5
(Partial alignment)
Sequences (1:14) Aligned. Score: 23.5
```

```
(Partial alignment)
Sequences (1:15) Aligned. Score: 16
(Partial alignment)
Sequences (2:3) Aligned. Score: 41.5
(Partial alignment)
Sequences (2:4) Aligned. Score: 41.5
(Partial alignment)
Sequences (2:5) Aligned. Score: 41.5
(Partial alignment)
Sequences (2:6) Aligned. Score: 41.5
(Partial alignment)
Sequences (2:7) Aligned. Score: 41.5
(Partial alignment)
Sequences (2:8) Aligned. Score: 41.5
(Partial alignment)
Sequences (2:9) Aligned. Score: 41.5
(Partial alignment)
Sequences (2:10) Aligned. Score: 41.5
(Partial alignment)
Sequences (2:11) Aligned. Score: 61.5
(Partial alignment)
Sequences (2:12) Aligned. Score: 61.5
(Partial alignment)
Sequences (2:13) Aligned. Score: 39.5
(Partial alignment)
Sequences (2:14) Aligned. Score: 23.5
(Partial alignment)
Sequences (2:15) Aligned. Score: 16
(Partial alignment)
Sequences (3:4) Aligned. Score: 41.5
(Partial alignment)
Sequences (3:5) Aligned. Score: 41.5
(Partial alignment)
Sequences (3:6) Aligned. Score: 41.5
(Partial alignment)
Sequences (3:7) Aligned. Score: 41.5
(Partial alignment)
Sequences (3:8) Aligned. Score: 41.5
(Partial alignment)
Sequences (3:9) Aligned. Score: 41.5
(Partial alignment)
Sequences (3:10) Aligned. Score: 41.5
(Partial alignment)
Sequences (3:11) Aligned. Score: 61.5
(Partial alignment)
Sequences (3:12) Aligned. Score: 61.5
(Partial alignment)
Sequences (3:13) Aligned. Score: 39.5
(Partial alignment)
Sequences (3:14) Aligned. Score: 23.5
(Partial alignment)
Sequences (3:15) Aligned. Score: 16
(Partial alignment)
Sequences (4:5) Aligned. Score: 41.5
(Partial alignment)
Sequences (4:6) Aligned. Score: 41.5
(Partial alignment)
Sequences (4:7) Aligned. Score: 41.5
(Partial alignment)
Sequences (4:8) Aligned. Score: 41.5
(Partial alignment)
Sequences (4:9) Aligned. Score: 41.5
(Partial alignment)
Sequences (4:10) Aligned. Score: 41.5
(Partial alignment)
Sequences (4:11) Aligned. Score: 61.5
(Partial alignment)
Sequences (4:12) Aligned. Score: 61.5
(Partial alignment)
Sequences (4:13) Aligned. Score: 39.5
```

```
(Partial alignment)
Sequences (4:14) Aligned. Score: 23.5
(Partial alignment)
Sequences (4:15) Aligned. Score: 16
(Partial alignment)
Sequences (5:6) Aligned. Score: 41.5
(Partial alignment)
Sequences (5:7) Aligned. Score: 41.5
(Partial alignment)
Sequences (5:8) Aligned. Score: 41.5
(Partial alignment)
Sequences (5:9) Aligned. Score: 41.5
(Partial alignment)
Sequences (5:10) Aligned. Score: 41.5
(Partial alignment)
Sequences (5:11) Aligned. Score: 61.5
(Partial alignment)
Sequences (5:12) Aligned. Score: 61.5
(Partial alignment)
Sequences (5:13) Aligned. Score: 39.5
(Partial alignment)
Sequences (5:14) Aligned. Score: 23.5
(Partial alignment)
Sequences (5:15) Aligned. Score: 16
(Partial alignment)
Sequences (6:7) Aligned. Score: 41.5
(Partial alignment)
Sequences (6:8) Aligned. Score: 41.5
(Partial alignment)
Sequences (6:9) Aligned. Score: 41.5
(Partial alignment)
Sequences (6:10) Aligned. Score: 41.5
(Partial alignment)
Sequences (6:11) Aligned. Score: 61.5
(Partial alignment)
Sequences (6:12) Aligned. Score: 61.5
(Partial alignment)
Sequences (6:13) Aligned. Score: 39.5
(Partial alignment)
Sequences (6:14) Aligned. Score: 23.5
(Partial alignment)
Sequences (6:15) Aligned. Score: 16
(Partial alignment)
Sequences (7:8) Aligned. Score: 41.5
(Partial alignment)
Sequences (7:9) Aligned. Score: 41.5
(Partial alignment)
Sequences (7:10) Aligned. Score: 41.5
(Partial alignment)
Sequences (7:11) Aligned. Score: 61.5
(Partial alignment)
Sequences (7:12) Aligned. Score: 61.5
(Partial alignment)
Sequences (7:13) Aligned. Score: 39.5
(Partial alignment)
Sequences (7:14) Aligned. Score: 23.5
(Partial alignment)
Sequences (7:15) Aligned. Score: 16
(Partial alignment)
Sequences (8:9) Aligned. Score: 41.5
(Partial alignment)
Sequences (8:10) Aligned. Score: 41.5
(Partial alignment)
Sequences (8:11) Aligned. Score: 61.5
(Partial alignment)
Sequences (8:12) Aligned. Score: 61.5
(Partial alignment)
Sequences (8:13) Aligned. Score: 39.5
(Partial alignment)
Sequences (8:14) Aligned. Score: 23.5
```

```
(Partial alignment)
Sequences (8:15) Aligned. Score: 16
(Partial alignment)
Sequences (9:10) Aligned. Score: 41.5
(Partial alignment)
Sequences (9:11) Aligned. Score: 61.5
(Partial alignment)
Sequences (9:12) Aligned. Score: 61.5
(Partial alignment)
Sequences (9:13) Aligned. Score: 39.5
(Partial alignment)
Sequences (9:14) Aligned. Score: 23.5
(Partial alignment)
Sequences (9:15) Aligned. Score: 16
(Partial alignment)
Sequences (10:11) Aligned. Score: 61.5
(Partial alignment)
Sequences (10:12) Aligned. Score: 61.5
(Partial alignment)
Sequences (10:13) Aligned. Score: 39.5
(Partial alignment)
Sequences (10:14) Aligned. Score: 23.5
(Partial alignment)
Sequences (10:15) Aligned. Score: 16
(Partial alignment)
Sequences (11:12) Aligned. Score: 84.3902
(Partial alignment)
Sequences (11:13) Aligned. Score: 61
(Partial alignment)
Sequences (11:14) Aligned. Score: 40.9756
(Partial alignment)
Sequences (11:15) Aligned. Score: 16.5854
(Partial alignment)
Sequences (12:13) Aligned. Score: 61
(Partial alignment)
Sequences (12:14) Aligned. Score: 40.9756
(Partial alignment)
Sequences (12:15) Aligned. Score: 16.5854
(Partial alignment)
Sequences (13:14) Aligned. Score: 35
(Partial alignment)
Sequences (13:15) Aligned. Score: 17.5
(Partial alignment)
Sequences (14:15) Aligned. Score: 23.6715
Guide tree file created:   [clustalw.dnd]

There are 14 groups
Start of Multiple Alignment

Aligning...
Group 1: Sequences:   2      Score:3496
Group 2: Sequences:   3      Score:3133
Group 3: Sequences:   4      Score:2180
Group 4: Sequences:   5      Score:2242
Group 5: Sequences:   6      Score:2271
Group 6: Sequences:   7      Score:2287
Group 7: Sequences:   8      Score:2298
Group 8: Sequences:   9      Score:2306
Group 9: Sequences:  10      Score:2312
Group 10: Sequences:  11      Score:2316
Group 11: Sequences:  12      Score:2319
Group 12: Sequences:  13      Score:2149
Group 13: Sequences:  14      Score:1750
Group 14: Sequences:  15      Score:1805
Alignment Score 114535

CLUSTAL-Alignment file created  [clustalw.aln]
```

clustalw.aln

```
CLUSTAL 2.1 multiple sequence alignment


NC_004718.3_SARS_coronavirus        ----------GGTGTTGGTACCTTCATGAAGGTCACC---AAACTGCTG-
AY274119.3_SARS-related_corona      ----------GGTGTTGGTACCTTCATGAAGGTCACC---AAACTGCTG-
LR757995.1_Wuhanvirus               ----------GTTGTTCGTTCTATGAAGACTTTTTAG---AGTATCATG-
MT192773.1_SARS-CoV-2/VNM           ----------GTTGTTCGTTCTATGAAGACTTTTTAG---AGTATCATG-
MN988713.1_Wuhanvirus               ----------GTTGTTCGTTCTATGAAGACTTTTTAG---AGTATCATG-
MT192772.1_SARS-CoV-2/VNM           ----------GTTGTTCGTTCTATGAAGACTTTTTAG---AGTATCATG-
MT163720.1¬_SARS-CoV-2/USA          ----------GTTGTTCGTTCTATGAAGACTTTTTAG---AGTATCATG-
MT163716.1¬_SARS-CoV-2/USA          ----------GTTGTTCGTTCTATGAAGACTTTTTAG---AGTATCATG-
MN996529.1_SARS-CoV-2/CHN           ----------GTTGTTCGTTCTATGAAGACTTTTTAG---AGTATCATG-
MN996528.1¬¬¬_SARS-CoV-2/VNM        ----------GTTGTTCGTTCTATGAAGACTTTTTAG---AGTATCATG-
MT012098.1_SARS-CoV-2/IND           ----------GTTGTTCGTTCTATGAAGACTTTTTAG---AGTATCATG-
LR757998.1_Wuhanvirus               ----------GTTGTTCGTTCTATGAAGACTTTTTAG---AGTATCATG-
MG772933.1_Bat_SARS-like_coron      ----------GGTGTTCGTATTATGAAGACTTTCTAG---AGTACCATG-
NC_014470.1_Bat_coronavirus         ----------TGTGTCCTATCTTTGCTACTTTTCTTGGTCATTATAATGC
KU740200.1_MERS/Egypt               GGTAATTACAGGAGTCCG-CCTATTACGGCGGATATT-----GAACTTG-
                                                :**       :* . .    : :        .   **

NC_004718.3_SARS_coronavirus        ------CATTT-AGAGACGTACTTGTTGTTTTAA---ATAAACGAACAAA
AY274119.3_SARS-related_corona      ------CATTT-AGAGACGTACTTGTTGTTTTAA---ATAAACGAACAAA
LR757995.1_Wuhanvirus               ------ACGTT-CGTGTTGTTTTAG--ATTTCAT---CTAAACGAACAAA
MT192773.1_SARS-CoV-2/VNM           ------ACGTT-CGTGTTGTTTTAG--ATTTCAT---CTAAACGAACAAA
MN988713.1_Wuhanvirus               ------ACGTT-CGTGTTGTTTTAG--ATTTCAT---CTAAACGAACAAA
MT192772.1_SARS-CoV-2/VNM           ------ACGTT-CGTGTTGTTTTAG--ATTTCAT---CTAAACGAACAAA
MT163720.1¬_SARS-CoV-2/USA          ------ACGTT-CGTGTTGTTTTAG--ATTTCAT---CTAAACGAACAAA
MT163716.1¬_SARS-CoV-2/USA          ------ACGTT-CGTGTTGTTTTAG--ATTTCAT---CTAAACGAACAAA
MN996529.1_SARS-CoV-2/CHN           ------ACGTT-CGTGTTGTTTTAG--ATTTCAT---CTAAACGAACAAA
MN996528.1¬¬¬_SARS-CoV-2/VNM        ------ACGTT-CGTGTTGTTTTAG--ATTTCAT---CTAAACGAACAAA
MT012098.1_SARS-CoV-2/IND           ------ACGTT-CGTGTTGTTTTAG--ATTTCAT---CTAAACGAACAAA
LR757998.1_Wuhanvirus               ------ACGTT-CGTGTTGTTTTAG--ATTTCAT---CTAAACGAACAAA
MG772933.1_Bat_SARS-like_coron      ------ACATT-CGTGTTGTCTTAG--ATTTCAT---CTAAACGAACTAA
NC_014470.1_Bat_coronavirus         TAATCATCTTT-TGTTTTGTGTTAG-AATTACAAGATCTAAACGAACAAT
KU740200.1_MERS/Egypt               -------CATTGCTTCGAGCTTAGG----CTCTT----TAGTAAGAGTAT
                                           . **   :   *   : *     : ::    **.:...* :*:

NC_004718.3_SARS_coronavirus        TTAAAATGTCTGATAATGGACCCCAATCAAACCAACGTAGTG-CCCCCCG
AY274119.3_SARS-related_corona      TTAAAATGTCTGATAATGGACCCCAATCAAACCAACGTAGTG-CCCCCCG
LR757995.1_Wuhanvirus               CTAAAATGTCTGATAATGGACCCCAA---AATCAGCGAAATG-CACCCCG
MT192773.1_SARS-CoV-2/VNM           CTAAAATGTCTGATAATGGACCCCAA---AATCAGCGAAATG-CACCCCG
MN988713.1_Wuhanvirus               CTAAAATGTCTGATAATGGACCCCAA---AATCAGCGAAATG-CACCCCG
MT192772.1_SARS-CoV-2/VNM           CTAAAATGTCTGATAATGGACCCCAA---AATCAGCGAAATG-CACCCCG
MT163720.1¬_SARS-CoV-2/USA          CTAAAATGTCTGATAATGGACCCCAA---AATCAGCGAAATG-CACCCCG
MT163716.1¬_SARS-CoV-2/USA          CTAAAATGTCTGATAATGGACCCCAA---AATCAGCGAAATG-CACCCCG
MN996529.1_SARS-CoV-2/CHN           CTAAAATGTCTGATAATGGACCCCAA---AATCAGCGAAATG-CACCCCG
MN996528.1¬¬¬_SARS-CoV-2/VNM        CTAAAATGTCTGATAATGGACCCCAA---AATCAGCGAAATG-CACCCCG
MT012098.1_SARS-CoV-2/IND           CTAAAATGTCTGATAATGGACCCCAA---AATCAGCGAAATG-CACCCCG
LR757998.1_Wuhanvirus               CTAAAATGTCTGATAATGGACCCCAA---AATCAGCGAAATG-CACCCCG
MG772933.1_Bat_SARS-like_coron      CTAAAATGTCTGATAATGGACCCCAA---AACCAACGTAGTG-CACCCCG
NC_014470.1_Bat_coronavirus         --AAAATGACTGATAATGGACAATCA---AACTCGCGTAATG-CGCCTCG
KU740200.1_MERS/Egypt               CTTAATTGATT-TTAACGAATCTCAA---TTTCATTGTTATGGCATCCCC
                                      :**:**: * :*** *.* .  .*   ::  .  *::.** *  * *

NC_004718.3_SARS_coronavirus        CATTACATTTGGTGGACCCACAGATTCAACTGACAATAACCAG--AATGG
AY274119.3_SARS-related_corona      CATTACATTTGGTGGACCCACAGATTCAACTGACAATAACCAG--AATGG
LR757995.1_Wuhanvirus               CATTACGTTTGGTGGACCCTCAGATTCAACTGGCAGTAACCAG--AATGG
MT192773.1_SARS-CoV-2/VNM           CATTACGTTTGGTGGACCCTCAGATTCAACTGGCAGTAACCAG--AATGG
MN988713.1_Wuhanvirus               CATTACGTTTGGTGGACCCTCAGATTCAACTGGCAGTAACCAG--AATGG
MT192772.1_SARS-CoV-2/VNM           CATTACGTTTGGTGGACCCTCAGATTCAACTGGCAGTAACCAG--AATGG
MT163720.1¬_SARS-CoV-2/USA          CATTACGTTTGGTGGACCCTCAGATTCAACTGGCAGTAACCAG--AATGG
MT163716.1¬_SARS-CoV-2/USA          CATTACGTTTGGTGGACCCTCAGATTCAACTGGCAGTAACCAG--AATGG
MN996529.1_SARS-CoV-2/CHN           CATTACGTTTGGTGGACCCTCAGATTCAACTGGCAGTAACCAG--AATGG
MN996528.1¬¬¬_SARS-CoV-2/VNM        CATTACGTTTGGTGGACCCTCAGATTCAACTGGCAGTAACCAG--AATGG
MT012098.1_SARS-CoV-2/IND           CATTACGTTTGGTGGACCCTCAGATTCAACTGGCAGTAACCAG--AATGG
LR757998.1_Wuhanvirus               CATTACGTTTGGTGGACCCTCAGATTCAACTGGCAGTAACCAG--AATGG
MG772933.1_Bat_SARS-like_coron      CATTACATTTGGTGGACCCTCAGATTCAAGTGACAATAGCAAA--AACGG
NC_014470.1_Bat_coronavirus         CATTACGTTTGGTG---TCTCAGATACCTCAGACAATAATCAG--AATGC
```

```
KU740200.1_MERS/Egypt             TGCTGCACCTCGTG------CTGTTTCCTTTGCCGATAACAATGATATAA
                                   . *.*.   * ***      *:*:*:*.: :* *..**. .*   :* .

NC_004718.3_SARS_coronavirus       AGGACGCAATGGGG-CAAGGCCAAAACAGCG---CC---
AY274119.3_SARS-related_corona     AGGACGCAATGGGG-CAAGGCCAAAACAGCG---CC---
LR757995.1_Wuhanvirus              AGAACGCAGTGGGG-CGCGATCAAAACAACG---TC---
MT192773.1_SARS-CoV-2/VNM          AGAACGCAGTGGGG-CGCGATCAAAACAACG---TC---
MN988713.1_Wuhanvirus              AGAACGCAGTGGGG-CGCGATCAAAACAACG---TC---
MT192772.1_SARS-CoV-2/VNM          AGAACGCAGTGGGG-CGCGATCAAAACAACG---TC---
MT163720.1¬_SARS-CoV-2/USA         AGAACGCAGTGGGG-CGCGATCAAAACAACG---TC---
MT163716.1¬_SARS-CoV-2/USA         AGAACGCAGTGGGG-CGCGATCAAAACAACG---TC---
MN996529.1_SARS-CoV-2/CHN          AGAACGCAGTGGGG-CGCGATCAAAACAACG---TC---
MN996528.1¬¬¬_SARS-CoV-2/VNM       AGAACGCAGTGGGG-CGCGATCAAAACAACG---TC---
MT012098.1_SARS-CoV-2/IND          AGAACGCAGTGGGG-CGCGATCAAAACAACG---TC---
LR757998.1_Wuhanvirus              AGAACGCAGTGGGG-CGCGATCAAAACAACG---TC---
MG772933.1_Bat_SARS-like_coron     AGAGCGCAATGGTG-CACGACCTAAACAACG---TC---
NC_014470.1_Bat_coronavirus        AGAACGTGCTGGAG-CGCGGCCAAAGCAAG---AA---
KU740200.1_MERS/Egypt              CAAATACAAACCTGTCTCGAGGTAGAGGACGTAATCCAA
                                   .... . . :   * * .*.  :*.. ...*    .
```

---

clustalw.dnd

```
(
(
(
(
(
(
LR757995.1_Wuhanvirus:0.29013,
(
NC_004718.3_SARS_coronavirus:0.07805,
AY274119.3_SARS-related_coronavirus:0.07805)
:0.01682)
:0.00074,
MT192773.1_SARS-CoV-2/VNM:0.29176)
:0.00042,
MN988713.1_Wuhanvirus:0.29208)
:0.00025,
MT192772.1_SARS-CoV-2/VNM:0.29225)
:0.00000,
MT163720.1¬_SARS-CoV-2/USA:0.29244)
:0.00000,
(
(
(
(
LR757998.1_Wuhanvirus:0.29118,
(
MG772933.1_Bat_SARS-like_coronavirus:0.25961,
(
NC_014470.1_Bat_coronavirus:0.32730,
KU740200.1_MERS/Egypt:0.43598)
:0.09625)
:0.04382)
:0.00015,
MT012098.1_SARS-CoV-2/IND:0.29235)
:0.00000,
MN996528.1¬¬¬_SARS-CoV-2/VNM:0.29241)
:0.00000,
MN996529.1_SARS-CoV-2/CHN:0.29245)
:0.00000,
MT163716.1¬_SARS-CoV-2/USA:0.29245);
```

Select tree menu ▼ Exec
