# Supplementary material for: Real-time reverse transcription loop-mediated isothermal amplification for rapid detection of SARS-CoV-2
Source: PeerJ. 2020 Jun 3;8:e9278. doi: 10.7717/peerj.9278 (PMC7275676; doi:10.7717/peerj.9278)
Supplement: Supplemental Information 5 — Note: Patent application filed (Patent application no: PI 2020002230). [file peerj-08-9278-s005.docx]

| Primers | Sequences (5’ to 3’) |
| --- | --- |
| F3 | GTTGTTCGTTCTATGAAGACT |
| B3 | GACGTTGTTTTGATCGCG |
| FIP | TGGGGTCCATTATCAGACATTTTAGTTTTAGAGTATCATGACGTTCG |
| BIP | CGAAATGCACCCCGCATTACCCACTGCGTTCTCCATTC |
| FLP | TGTTCGTTTAGATGAAATC |
| BLP | TGGTGGACCCTCAGATTCAA |

**Supplementary Table 1:** RT-LAMP primers used in this study.

Note: Patent application filed (Patent application no: PI 2020002230).
